# Livestock Density as Risk Factor for Livestock-associated Methicillin-Resistant *Staphylococcus aureus*, the Netherlands

**DOI:** 10.3201/eid1909.130876

**Published:** 2013-09

**Authors:** Beth J. Feingold, Jan A.J.W. Kluytmans, Brigite A.G.L. van Cleef, Frank C. Curriero, Ellen K. Silbergeld

**Affiliations:** Johns Hopkins Bloomberg School of Public Health, Baltimore, Maryland, USA (B.J. Feingold, F.C. Curriero, E.K. Silbergeld);; Amphia Hospital, Breda, the Netherlands (J.A.J.W. Kluytmans, B.A.G.L. van Cleef);; St. Elisabeth Hospital, Tilburg, the Netherlands (J.A.J.W. Kluytmans, B.A.G.L. van Cleef);; VU University Medical Center, Amsterdam, the Netherlands (J.A.J.W. Kluytmans, B.A.G.L. van Cleef)

**Keywords:** swine, livestock, cattle, geographic information systems, methicillin-resistant Staphylococcus aureus, MRSA, cluster analysis, bacteria, zoonoses, the Netherlands

**In Response:** We thank Davies et al. for their letter (*1*) responding to our report (*2*). We appreciate the opportunity to address their comments, some of which raise appropriate concerns.

Davies et al. correctly note that the original methicillin-resistant *S. aureus* (MRSA) registry data were derived from swab specimens and clinical isolates; the distribution of anatomic sites differed between the cases and controls in our study. However, all study participants were proven MRSA carriers. As per their comment in their letter (*1*), we reran multivariate models excluding 5 persons who acquired MRSA outside the Netherlands. We found odds ratios (and p<0.05) similar to those originally reported for covariates of municipality-level livestock densities.

Davies et al. apparently pooled data from 3 studies and stated that there is negligible risk for livestock-associated–MRSA among persons who do not have livestock or farm contact. Each of these studies was designed differently and had different comparison groups, and each report conceded that factors such as indirect human or environmental transmission could have exposed study participants who lacked known farm risk factors.

In addition, Davies et al. state that our observed association of increased odds of livestock-associated–MRSA compared with a typeable strain of MRSA in regions with higher livestock densities should be limited to hypothesis generation. We agree, as stated in the final sentence of our report (*2*). However, a similar association was confirmed in a recent study in the Netherlands that included MRSA-negative controls (*3*).

To clarify our statement comparing pig densities in the United States and the Netherlands (*2*), we referred to density in terms of animals per operation, a relevant parameter for other zoonotic diseases, including swine and avian influenzas. To state this information in a different manner, in 2007, a total of 60% of the 67.7 million pigs raised in the United States were raised on farms with >5,000 pigs, but only 22% of the 11.66 million pigs raised in the Netherlands were raised on farms with >5,000 pigs (*4*,*5*).
